# Fatal intramyocardial dissecting haematoma caused by coronary artery perforation during percutaneous coronary intervention

**DOI:** 10.1093/ehjimp/qyae030

**Published:** 2024-04-23

**Authors:** Hiroki Yamanobe, Kensaku Nishihira, Mitsuhiro Yano, Yoshisato Shibata, Yujiro Asada

**Affiliations:** Department of Cardiology, Miyazaki Medical Association Hospital, 1173 Arita, Miyazaki, Miyazaki 880-2102, Japan; Department of Cardiology, Miyazaki Medical Association Hospital, 1173 Arita, Miyazaki, Miyazaki 880-2102, Japan; Department of Cardiovascular Surgery, Miyazaki Medical Association Hospital, Miyazaki, Japan; Department of Cardiology, Miyazaki Medical Association Hospital, 1173 Arita, Miyazaki, Miyazaki 880-2102, Japan; Department of Diagnostic Pathology, Miyazaki Medical Association Hospital, Miyazaki, Japan

**Keywords:** autopsy, coronary artery perforation, histopathology, intramyocardial dissecting haematoma

A 75-year-old man with atrial fibrillation was hospitalized due to angina pectoris and underwent percutaneous coronary intervention (PCI) for severe stenosis in the middle segment of the right coronary artery at another hospital (arrows; *Figure 1A1* and Supplementary data online, *Video S1*). The patient had received antiplatelet therapy with aspirin (100 mg/day) and clopidogrel (75 mg/day) plus anticoagulation with apixaban (10 mg/day). During PCI, a coronary wire strayed into the far distal branch (*Figure 1A2* and Supplementary data online, *Video S2*), resulting in coronary artery perforation (*Figure 1A3* and Supplementary data online, *Videos S3* and *S4*). Echocardiography revealed pericardial effusion (see Supplementary data online, *Video S5*). Despite haemostasis with coil embolization and pericardial drainage for cardiac tamponade, the patient was transferred to our hospital because of persistent oozing bleeding and haemodynamic instability. At this point, we suspected cardiac rupture. After discussions within our heart team, we performed emergency surgical left ventricular repair. Intraoperatively, an extensive haematoma was observed from the inferolateral wall to the apex of the heart, consistent with the presumed site of coronary artery perforation (*Figure 1B*). Five days after surgery, he died of multiple organ failure. Autopsy showed intramyocardial dissection in the superficial layer of the left ventricle, which was filled with a large haematoma (asterisks, *Figure 1C1* and *C2*). Necrotic cardiomyocytes were observed close to the haematoma (*Figure 1C3*). Thus, the patient was diagnosed with intramyocardial dissecting haematoma associated with coronary artery perforation during PCI.

**Figure 1:**
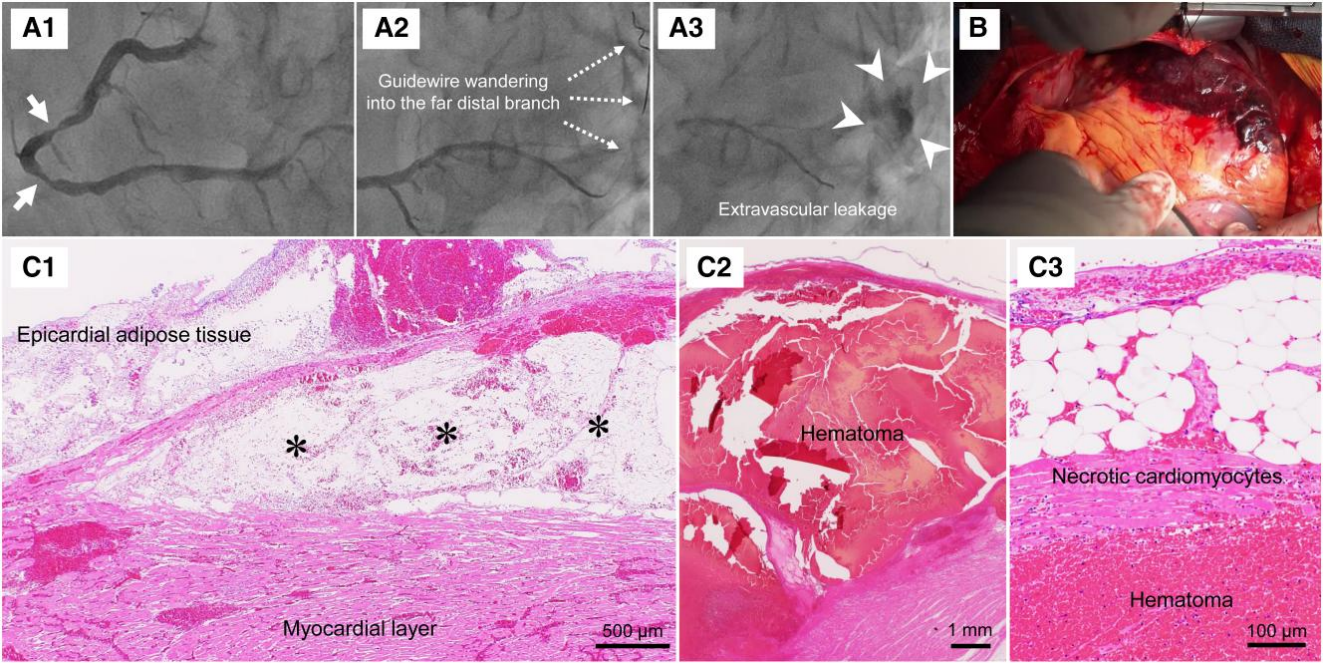
(*A1*) Pre-procedural coronary angiography. Coronary angiography showed severe stenosis in the right coronary artery (arrows). (*A2*) Guidewire wandering into the far distal branch. (*A3*) Leakage of contrast media due to coronary artery perforation (arrowheads). (*B*) Operative findings. (*C1*–*C3*) Histopathology of autopsy specimens. Dissected myocardium (asterisks) and haematoma were observed.

Intramyocardial dissecting haematoma is a subtype of cardiac rupture in which helical dissection of myocardial fibre layers results in partial rupture. It has been recently reported to occur in 0.3% of patients with ST-segment elevation myocardial infarction, but the frequency attributed to PCI is unknown. We should be aware of this very rare but possible fatal complication of PCI.

## Supplementary data

Supplementary data are available at *European Heart Journal – Imaging Methods and Practice* online.

**Funding:** None declared.

**Consent:** The authors confirm that written consent for submission and publication of this case report, including images and associated text, has been obtained from the patient’s family.

**Data availability:** No new data were generated or analysed in support of this article.

## Lead author biography


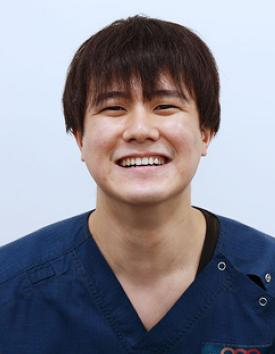


Dr Hiroki Yamanobe graduated from the Faculty of Medicine, Chiba University, in 2021. Currently, he works as a resident in the cardiology department at Miyazaki Medical Association Hospital. He has a special interest in heart failure and intensive cardiac care.

## Supplementary Material

qyae030_Supplementary_Data

